# Dislocation of the Shoulder

**Published:** 1847-01

**Authors:** 


*Dislocation of the Shoulder*—M. Robert exhibited to the meeting of the Society of Surgery a dislocation of the humerus downwards, artificially produced on the dead subject. The head of the bone rests upon the ridge of the scapula, immediately beneath the glenoïd cavity, in a sort of groove formed in front by the subscapularis, and posteriorly by the long portion of the triceps muscle. The form of displacement had been admitted by T. L. Petit, Boyer, A. Cooper, &c., and M. Malgaigne, in refusing to admit its possibility, had, M. Robert believed, been guilty of error.

M. Malgaigne insisted that M. Robert's artificial luxation was not downwards only, but also forward; the head of the humerus being on the inner side of a line drawn perpendicularly from the glenoïd surface.

M. Chassaignac had collected twelve *post-mortem* examinations of recent dislocation of the shoulder; the head of the humerus was most generally found to lie on the anterior face of the scapula, inside the glenoïd cavity; and at a variable distance from the coracoïd process. Sometimes it may rise as high as the basis of that bony projection; at other times, it is seated below the glenoïd surface ; and in some cases rests upon the axillary margin of the scapula. These differences of position, M. Chassaignac had long since mentioned in his annotations to the translation of Sir A. Cooper's works ; he considered them to be of importance, inasmuch as they explain very material differences in the signs of the displacement.—*Ibid.*

